# Why was PERV not transmitted during preclinical and clinical xenotransplantation trials and after inoculation of animals?

**DOI:** 10.1186/s12977-018-0411-8

**Published:** 2018-04-02

**Authors:** Joachim Denner

**Affiliations:** Robert Koch Institute, Nordufer 20, 13353 Berlin, Germany

## Abstract

Porcine endogenous retroviruses (PERVs) are present in the genome of all pigs, they infect certain human cells and therefore pose a special risk for xenotransplantation using pig cells, tissues and organs. Xenotransplantation is being developed in order to alleviate the reduced availability of human organs. Despite the fact that PERVs are able to infect certain human cells and cells from other species, transmission of PERVs has not been observed when animals (including non-human primates) were inoculated with PERV preparations or during preclinical xenotransplantations. The data indicate that PERVs were not transmitted because they were not released from the transplant or were inhibited by intracellular restriction factors and innate immunity in the recipient. In a single study in guinea pigs, a transient PERV infection and anti-PERV antibodies were described, indicating that in this case at least, the immune system may also have been involved.

## Xenotransplantation: the need, the problems and the progress

Xenotransplantation using pig cells, tissues and organs is being developed in response to the steadily decreasing availability of human organs and due to an increased need by the aging human population [1]. In the US, 114,965 people are in desperate need of a lifesaving organ transplant (total waiting list candidates) and of those, 74,816 people are active waiting list candidates [2]. In contrast, only 2853 transplantations were performed in January 2018. On average, 20 people die each day while waiting for a transplant. Xenotransplantation using pig islet cells may be also the most effective solution for the treatment of diabetes. In 2015, 30.3 million Americans, or 9.4% of the population, had diabetes, among them 1.25 million American children and adults with type 1 diabetes [3]. Although type 1 diabetes can be treated with insulin, complications including limb amputations and blindness due to poor patient compliance are the main cost factors when treating the disease. Pig islet cells producing insulin under biological regulation may therefore be the better solution.

Pigs, for several reasons including similar physiology, size, low costs as well as the ability to be cloned and easily genetically modified, are the most suitable donor animals. Pig insulin was used for decades to treat diabetes until recombinant human insulin became available. Although there are several barriers to successful clinical xenotransplantation including immunological rejection, physiological incompatibility and the risk of transmission of porcine microorganisms to the human xenotransplant recipient, significant progress has been made in recent years [4, 5]. The problem of immunological rejection can be solved by multiple genetic modifications in the pigs and a more effective immunosuppression [6, 7]. As a result, the recently measured survival times of pig organ transplants in non-human primates are impressive: pig islet cells can maintain insulin-independent normoglycemia for up to 950 days in diabetic monkeys [8] and the best survival time for the heterotopic transplantation of pig hearts to non-human primates has increased to 945 days [9]. Kidney transplantats have survived for 6-months [10–12] and a maximum survival of 90 days in orthotopic heart transplantation has been reported recently [13].

However, there is still the risk of transmission of porcine microorganisms to the human recipient. Some potentially zoonotic viruses have been well studied, and sensitive detection methods as well as elimination programs have been developed. Among these viruses are the porcine cytomegalovirus (PCMV, for review see [14, 15]), the hepatitis E virus (HEV, for review see [16, 17]), the porcine lymphotropic herpesviruses [18, 19] and the porcine circoviruses [20, 21]. An analysis of the porcine virome revealed many other viruses [22]. PCMV was shown to reduce significantly the survival time of pig kidney transplants in non-human primates (NHP) [15, 23, 24]. HEV is a well-known zoonotic virus which can be transmitted to humans by undercooked pork meal or contact with pigs. HEV induces chronic infections in immunosuppressed patients and severe liver diseases in patients with pre-existing liver failure [16, 17].

## Porcine endogenous retroviruses (PERVs)

Whereas most porcine viruses, bacteria and fungi can be eliminated by selection of negative animals, vaccination, treatment, early weaning, Caesarean delivery or embryo transfer, this is impossible in the case of PERVs [25–27]. PERV-A and PERV-B are integrated as DNA copies (proviruses) in the genome of all pigs and PERV-C is found in most but not all pigs [28]. PERV-A, -B, and -C are gammaretroviruses, the porcine endogenous betaretroviruses are not well studied [29, 30]. PERV-A, -B, and -C are closely related to the murine leukaemia virus (MuLV), the feline leukaemia virus (FeLV) and the koala retrovirus (KoRV) [28]. The related MuLV, FeLV and KoRV like many other retroviruses induce tumours and immunodeficiencies associated with opportunistic infections in the infected host (for review see [31–33]). Therefore the transmission of PERV to the human xenotransplant recipient could result in tumours and/or an immunodeficiency.

Pig cells can release virus particles able to infect cells from different species including humans (Table 1) [28, 34–46]. The number of PERV proviruses is different in different pig breeds, ranging from one to over a hundred (for review see [47]. There is evidence for de novo infections and/or transpositions of PERVs in the pig, leading to different copy numbers in different organs of an individual pig [47]. In addition, recombinations between PERV-A and PERV-C have been described in pigs and such PERV-A/C recombinants are similar to PERV-A in their ability to infect human cells but were shown to have a higher replication rate compared with PERV-A [48]. PERVs-A/C were found integrated in somatic pig cells, but not in the germ line. PERV-C is an ecotropic virus infecting only pig cells.

**Table 1:** PERV infection experiments using cultured cells of different species

Type of infection	Species	References
Productive infection with replication^a^	Immortalised human cells (e.g., 293 cells), cat, mink	[28, 34–39]
Infection without replication^b^	Primary human cells (e.g., PBMCs^d^, PAEC), rhesus monkey, baboon, gorilla, chimpanzee^d^	[28, 35, 37–43]
Absence of infection^c^	Mouse, rat, rabbit, cotton rat, horse, pig-tailed macaque, African green monkeys, cynomolgus monkeys	[28, 38, 44–46]

^a^Release of more virus particles than used for infection

^b^Release of less virus particles than used for infection

^c^Absence of provirus integration

^d^Using human-adapted PERV

## Conditions of PERV infection in cell culture

As mentioned above, PERV-A and PERV-B are polytropic viruses able to infect human cells and cells of other species (Table 1) [28, 34–43]. To understand the risk posed by PERV it is important to analyse which cells can be infected and under which conditions and whether this infection is productive, e.g., whether the virus replicates in the infected cells.

Two multi-membrane-spanning receptors have been described for PERV-A in humans initially named human porcine endogenous retrovirus A receptor 1 and 2 (huPAR-1, huPAR-2) [49]. Two similar receptors were also found in pigs [49]. These were subsequently shown to be members of the human riboflavin transporter family, hRFT3 and hRFT1, respectively, although they have since been renamed and classified as members of the solute carrier family 52A [50]: SLC52A1 corresponds to huPAR2 and SLC522 to huPAR1. Glycosylation of huPAR2 is not necessary for the PERV-A receptor function, but three cysteines play a critical role during infection [51].

HuPAR1 is fully functional as a viral receptor on human cells, but a variant receptor PAR1(109Ser-Leu) was found in NHP (baboons, rhesus monkeys, cynomolgus macaques), allowing only a limited infection [49, 52]. Although the receptor in African green monkeys is not different from the human receptor at position 109, PERV infection is still poor. The receptor in marmosets is also equal to that of humans but it is unknown whether it is functional [53]. The receptor on murine cells is also a variant and is not functional [54]. In the case of rat cells the amount of the receptor on the cell surface is normally too low to facilitate infection, although copies increasing the receptor density by transfection rendered the cell permissive [54]. Transgenic mice expressing the human PERV-A receptor huPAR2 have been generated and after inoculation with infectious supernatant, viral DNA, RNA, protein and virus particles were detected in their organs, indicating productive viral infection [55]. However, follow-up studies showing a pathogenic effect of PERV infection have been not published.

The absence of infection in some cells can therefore be easily explained by the absence of a functional receptor [49, 52, 54] or by a suboptimal density of the receptor on the cell surface [54]. PERV-A and PERV-B easily infect human embryonic kidney 293 cells and this is a productive infection with the virus replicating and producing excess virus particles. Other human cells such as C8166, can also be infected, although it is unclear whether the infection is productive, i.e., whether virus particles were produced, because only provirus integration was demonstrated [40]. 293 cells are immortalised cells which have been shown to express a reduced number of intracellular restriction factors such as the apolipoprotein B mRNA editing enzyme catalytic (APOBEC) protein family [56]. Since human primary cells contain functional restriction factors (see below) it was difficult to infect them with PERV. Infection of human PBMCs was only achieved, when human cell-adapted viruses were used [57]. Human adapted viruses had been generated either from PERV-A/C recombinants isolated from pig lymphocytes or from PERV-A by serial passage on human 293 cells. Human cell-adapted viruses are characterised by an increased replication rate and genetic modifications in the long terminal repeats [40, 46, 58]. Other human primary cells [endothelial cells, vascular fibroblast, mesangial cells and porcine aorta endothelial cells (PAEC)] were successfully infected with PERV released directly from PK-15 cells [59], a pig kidney cell line producing low amounts of PERV particles. In that report it was shown that the infection was productive, as reverse transcriptase activity was observed in the supernatant of infected cells. Recently, infection of human umbilical vein endothelial cells (HUVEC) has been reported [60], although in this case it remains unclear whether this was a productive infection, or whether only the integrated provirus or even unintegrated proviral DNA was detected by PCR.

## PERVs and cellular restriction factors

As shown above, cellular restriction factors play an important role in preventing PERV infections. This is nicely demonstrated by the fact that 293 cells, which are most susceptible to PERV infection, do not express APOBEC3G. In contrast, primary cells expressing APOBEC3G and other restriction factors are difficult to infect [57]. APOBEC proteins are cytidine deaminases that disrupt viral DNA during synthesis. These deaminases cause G-to-A hypermutation in nascent retroviral DNA strands during reverse transcription. PERV transmission from virus-producing pig PK-15 cells to human cells was significantly reduced when human APOBEC3G, but not the porcine APOBEC3G, was expressed in PK-15 cells [61]. This inhibition did not require the DNA deaminase activity of APOBEC3G. Other studies showed that both human and porcine APOBEC3 are inhibitors of PERV [62]. Porcine and human APOBEC3 (A3) could inhibit PERV replication, thereby reducing the risk of infection of human cells by PERV [63]. The replication of PERVs in cells co-expressing human APOBEC3 s was reduced by 60–90% compared with PERV-only control [64]. PERV-B is severely inhibited by huA3G and porcine A3Z2-Z3 (poA3F) and PERV-C infectivity was strongly inhibited by poA3Z2-Z3, which did not markedly reduce PERV-B infectivity [65]. When in addition to APOBEC3G two other major classes of retroviral restriction factors, tetherin, and TRIM5α, were analysed, the antiviral activity of human tetherin was slightly weaker than that of human APOBEC3G (hA3G) [66]. A combination of tetherin and hA3G was more potent than each individual restriction factor. TRIM5a is a member of the tripartite motif (TRIM) protein family involved in diverse cellular processes. Although TRIM5a is highly effective in inhibiting HIV-1 and other retroviruses, PERV-A and PERV-A/C were insensitive to restriction by TRIM5a in feline cells expressing TRIM5a from humans, African green monkeys, rhesus macaques, squirrel monkeys, rabbits or cattle [67]. Tetherin is a type I interferon-inducible molecule that blocks release of retroviruses from infected cells. Overexpression of either human or porcine tetherin on pig cells significantly reduced PERV production [68]. Another restriction factor inhibiting PERV infection of human cells is sterile alpha motif and histidine-aspartate domain 1 containing protein (SAMHD1), a cellular enzyme with phosphohydrolase activity, converting deoxynucleoside triphosphates (dNTPs) to inorganic phosphate (iPPP) and a 2′-deoxynucleoside (i.e., deoxynucleosides without a phosphate group). SAMHD1 depletes the pool of dNTPs available to a reverse transcriptase for viral cDNA synthesis and thus prevents viral replication [69]. SAMHD1 was shown to inhibit infection of primary human monocytes, monocyte-derived dendritic cells and monocyte-derived macrophages with a human-cell adapted PERV-A/C (Al-Shehabi, H., Fiebig, U. Denner, J., Bannert N., Hofmann, H., in preparation).

Recently novel cellular restriction factors implicated in HIV-1 replication have been described [70] and it has to be analysed whether these proteins or other factors still unknown may also inhibit PERV.

## Absence of PERV transmission after inoculation of small laboratory animals and non-human primates

In order to establish an animal model system to study transmission and potential pathogenic effects, PERV infection experiments in small laboratory animals as well as in NHP were performed (Table 2) [39, 41, 42, 71]. In all but one of these experiments the failure to detect viral genomes by PCR and the lack of PERV-specific antibodies indicated that no infection has occurred. In agreement with the in vitro infection data (Table 1), mice, rats and NHP could not be infected due to an incompatible receptor that only allows a limited infection or because of the low density of a functional viral receptor. The absence of antibodies in these experiments indicated that there was either no infection at all or an infection at a level insufficient to induce an antibody response. This supports the suggestion that either the virus load was too low to overcome intracellular restriction factors or that other mechanisms of innate immunity were predominantly involved in the prevention of infection. In only a single case was a transient infection observed in Guinea pigs, with provirus being detected in different organs but disappearing after 16 weeks [71]. Either tightly controlled suppression of virus replication or a potent host clearance mechanism against PERV may explain the reduced levels of viral DNA detected at later time points. The latter interpretation is supported by the durable humoral immunity observed in these animals during the time-course of the experiment (16 weeks) [71].

**Table 2:** PERV inoculation experiments into small animals and NHP

Recipient	Virus source	Immuno-suppression, treatment	PERV testing		References
			PCR analysis	Antibody detection	
SCID mice^a^	Human cell-adapted PERV	None	Negative	nt	Irgang et al. [45]
	Transplantation of pig PBMCs	None	Negative	nt	Kuddus et al. [72]
Rats	Supernatant PK-15 cells, supernatant PERV-infected 293 cells, human cell-adapted PERV	Cyclosporine A, cobra venom factor	Negative	Negative	Denner et al. [73]
Mink	Supernatant PERV-infected 293 cells, human cell-adapted PERV	None	Negative	Negative	Specke et al. [39]
Guinea pigs	Supernatant PK-15 cells, supernatant PERV-infected 293 cells	None	Negative	Negative	Specke et al. [44]
	PERV-NIH	None	Transient positive	Positive	Argaw et al. [71]
Rhesus monkeys, pig-tailed monkeys, baboons	Human cell-adapted PERV	Cyclosporine A, everolimus (RAD), methyl-prednisolone	Negative	Negative	Specke et al. [41, 42]

*Nt* not tested

^a^Reports showing that SCID mice were infected with PERV [74, 75] were the result of an artefact based on pseudotyping between PERV and endogenous murine retroviruses [76, 77]

## Absence of PERV transmission in preclinical transplantations of different pig organs into non-human primates

In a recent review the setting and the results of seven preclinical trials involving 101 different non-human primates and transplanting pig hearts, kidneys, skin, islet cells and livers were analysed in detail (see [28]). None of the animals were infected with PERV. In the meantime, additional preclinical trials have been performed and analysed and these also show the absence of PERV transmission either by PCR or by Western blot analysis (Table 3) [53, 78–82]. However, keeping in mind, that the PERV receptor in NHP is not fully functional and the infection of NHP cells in vitro is not productive, this lack of infection in vivo is not surprising.

**Table 3:** Absence of PERV transmission in recent preclinical xenotransplantations

Donor pigs	Transplant	Recipient (number)	Immuno-suppression, encapsulation	PERV testing		References
				PCR analysis	Antibody detection	
Genetically modified large white × landrace or miniature swine	Heterotopic heart, kidney, thymokidney	Baboons (10)	Mycophenolate mofetil, FK506, anti-CD154mAb, anti-CD2mAb, steroids, radiation, anti-thymocyte globulin, cobra venom factor	Micro-chimerism	Nt	Issa et al. [78]
Genetically modified German landrace × large white	Orthotopic heart	Baboons (6)	Anti-CD20mAb, anti-CD40mAb, ATG, mycophenolate mofetil, methylprednisolone	Negative	Negative	Morozov et al. [79], Denner et al. unpublished
German landrace expressing *INS*LEA29Y	Islet cells	Marmosets (4)	None	Negative	Negative	Plotzki et al. [53]
Göttingen minipigs	Islet cells	Cynomolgus monkeys (8)	Macrodevice	Negative	Negative	Morozov et al. [80]
Large white × Yorkshire × landrace	Islet cells	Cynomolgus monkeys (6)	Agarose encapsulation	Negative	Nt	Gazda et al. [81]

Only recent trials and only trials performing PERV testing. Seven other trials have been analysed previously [28]

*Nt* not tested

## Absence of PERV transmission in clinical transplantations to humans

Several clinical trials have been performed in the past, transplanting islet cells for the treatment of diabetes, performing ex vivo perfusion using pig spleens or livers and transplanting neuronal cells (more than 200 cases, for review see [28]). PERV transmission has not been observed in any of the patients. However it is important to note, that in these trials no immunosuppression (or only a weak immunosuppression in the case of combined allogenic kidney and porcine islet cells transplantations [82]) was applied.

Recently, two clinical trials have been performed using pig islet cells to treat diabetes in humans in New Zealand and Argentina. In all cases a positive medical effect was observed [83, 84], and neither PERVs nor other porcine viruses under investigation were transmitted [85, 86]. Islet cells from Auckland island pigs were used for these studies. These animals were well characterised [87] and had been used in a prospective preclinical trial in cynomolgus monkeys during which PERV transmission was also not observed in this trial [88]. In addition, no pharmaceutical immunosuppression was applied because the islet cells were encapsulated. It has been shown that encapsulation prevents PERV release [89] and, furthermore, there is evidence that pig islet cells do not release PERV particles [90].

## Conclusion and perspectives

PERV transmission has not been observed in any of the many preclinical and clinical xenotransplantation trials performed so far, and not in any of the numerous experimental PERV infection experiments. Most of the clinical trials performed involved transplantations of pig cells, mainly encapsulated islet cells, in most cases without pharmaceutical immunosuppression. Due to the lack of functional PERV receptors in the NHP and small animal recipients, most of these experiments are not relevant for evaluating the potential risk to humans.

The risk posed by PERVs during xenotransplantation of pig tissues and organs is therefore difficult to evaluate based on these results. Transplanting vascularised large organs requires a strong immunosuppression, the organ cannot be encapsulated and usually cells of the blood and immune system will also be transmitted. Unfortunately, there is no way to definitively and reliably assess the risk posed by PERV experimentally: only long-term follow up of actual xenotransplant recipients will provide the answer.

To prevent PERV transmission after xenotransplantation, a range of different strategies have been developed, including selection of PERV-C free animals to prevent recombination between PERV-A and PERV-C [91, 92], selection of animals with a low expression of PERV-A and PERV-B [93], generation of transgenic pigs expressing a PERV-specific small-interfering (si) RNA that reduces the expression of PERV [94–98], development of a vaccine based on neutralising antibodies against the envelope proteins of PERV [99–102] and finally gene editing to inactivate all proviral copies in the genome using either a zinc finger nuclease [103] or the CRISPR/Cas9 technology [104, 105].

The successful generation of live piglets in which PERVs are inactivated using the CRISPR/Cas9 technology [60] will reduce the risk of PERV transmission to zero and raises the question of whether all donor pigs used for xenotransplantation should be derived from such a stock [60, 106–108].
